# The Diversity of Bacterial Symbionts in Major Agricultural Lepidopteran Pests in Australia

**DOI:** 10.1155/ijm/6897546

**Published:** 2026-07-13

**Authors:** Hareem Qazi, Joshua A. Thia, Jing Zhao, Samantha Edley, Paul A. Umina, Jasmeen Kaur, Kym D. Perry, Simon W. Baxter, Ary A. Hoffmann, Qiong Yang

**Affiliations:** ^1^ Bio21 Institute, School of BioSciences, The University of Melbourne, Parkville, Victoria, Australia, unimelb.edu.au; ^2^ Institute of Plant Protection, Jiangsu Academy of Agricultural Sciences, Nanjing, China, jaas.ac.cn; ^3^ Agriculture Victoria Research, Department of Energy Environment and Climate Action (DEECA), Mildura, Victoria, Australia; ^4^ Cesar Australia, Brunswick, Victoria, Australia; ^5^ School of Agriculture, Food and Wine, Adelaide University, Adelaide, Australia, adelaide.edu.au; ^6^ School of BioSciences, The University of Melbourne, Parkville, Victoria, Australia, unimelb.edu.au

**Keywords:** bacterial symbionts, endosymbionts, *Wolbachia*, *Enterococcus mundtii*, grain crops, lepidoptera, MLST, *Rickettsia*, *Spiroplasma*, survey

## Abstract

Lepidopteran pests are among the most destructive insects in agriculture, causing major yield losses across a wide range of crops worldwide. With growing emphasis on reducing chemical pesticide use, there is increasing interest in understanding their associated microbiomes and endosymbiotic bacteria. These microbial associates can influence host physiology, fitness, and pesticide/thermal resistance traits, and may provide novel opportunities for developing sustainable pest management strategies. Here, we characterized the diversity and distribution of various endosymbionts and other microbiota in agriculturally important lepidopteran pest species in Australia. In total, we screened 21 field‐collected and one commercially reared species of Lepidoptera. Our results indicate that endosymbiont infections were relatively rare and polymorphic in Australian Lepidoptera species. *Wolbachia* was the most prevalent endosymbiont in field‐collected and commercially available pest species. This symbiont can manipulate host reproduction through mechanisms such as cytoplasmic incompatibility and sex‐ratio distortion, with potential relevance for pest control. Our MLST analysis suggested that the *Wolbachia* belonged to Supergroups A and B. Targeted microbiota were present across various species, with a particularly high abundance of *Enterococcus mundtii* in *Spodoptera frugiperda* (Smith); this gut bacterium may play a role in pesticide resistance. Distance‐based compositional principal component analysis conducted on selected species (*Plutella xylostella* and *Helicoverpa spp*.) showed that there was no significant difference in bacterial composition between the *Helicoverpa species*, or between the host crops/geographic locations where these taxa were collected. These findings provide a basis for future investigations on the phenotypic effects of endosymbionts and gut microbiota in local pests, with long‐term potential to suppress pest populations sustainably.

## 1. Introduction

The order Lepidoptera (butterflies and moths) is one of the most diverse insect orders, with over 157,000 known species [1], including some of the most important agricultural pests worldwide [2]. At present, damage caused by invasive insect pests costs the global economy around $70 billion annually [3]. Many species of Lepidoptera are expanding their geographical ranges due to international trade and climate change [4–7], including major pest species, for example, diamondback moth (*Plutella xylostella*) [8–10], cotton bollworm (*Helicoverpa armigera*) [11–13] and fall armyworm (*Spodoptera frugiperda*) [14–16], which have become global agricultural pests. Lepidopteran pests of Australian agriculture include both native and introduced species [15, 17–19].

In Australian grain and horticulture industries, *P. xylostella*, *H. armigera* and *S. frugiperda* have become challenging to manage because of their resistance to chemical pesticides. In *P. xylostella* and *H. armigera*, resistance has evolved locally under strong selection pressure [20–22]. In contrast, *S. frugiperda* likely invaded already carrying pre‐existing resistance traits from its native range [23]. The growing issue of resistance and ongoing difficulties in controlling these pests have prompted efforts to explore alternative methods for their sustainable management.

Insects carry diverse bacterial microbial communities occupying different niches in their body, including intracellular endosymbionts, gut bacteria and cuticular microbiota [24, 25]. Of these, gut microbes and endosymbionts are the most widely studied, with increasing interest in their potential for use in pest control. *Wolbachia* and *Spiroplasma* are the most common endosymbionts in Lepidoptera; however, compared with other arthropod groups such as the Diptera, Hymenoptera and Hemiptera, overall endosymbiont diversity in Lepidoptera appears relatively low, with endosymbionts that are common in other insects, such as *Arsenophonus*, *Cardinium*, *Hamiltonella* and *Rickettsia,* being largely absent [26]. Nevertheless, there is a diversity of gut symbionts present in Lepidoptera [27].

Endosymbionts are typically maternally transmitted and can affect host reproduction, involving phenomena like male killing [28], parthenogenesis [29], feminization [30] and cytoplasmic incompatibility [31]. Although endosymbiont‐induced reproductive effects have been observed in various Lepidoptera species, as reviewed in Duplouy and Hornett [26], their direct impact on population suppression remains poorly documented [32]. However, successful use of *Wolbachia*‐induced cytoplasmic incompatibility in mosquitoes [33, 34], and its potential application in other pests such as plant hoppers [35], points to the promise of such strategies for pest control [36–39]. Such strategies can be used as a valuable tool for controlling sexually reproducing insect pests if harnessed effectively [35, 40, 41].

In addition to its well‐established effects on host reproduction, *Wolbachia* may also have broader relevance to pesticide resistance research. *Wolbachia* has been reported to upregulate P450 gene expression and indirectly enhance insecticide resistance in some host insects [42, 43], while offering potential applications in future biological control strategies. While endosymbionts are typically maternally inherited, gut microbiota in various insects, including Lepidoptera, are usually acquired horizontally and play an important role in improving digestion, immunity, host fitness and chemical detoxification [27, 44, 45]. In contrast to many other insect orders, lepidopteran gut microbiota are often transient and highly variable, with communities largely acquired from the environment and influenced by diet [46, 47]. Several gut bacterial genera such as *Achromobacter*, *Bacillus*, *Burkholderia*, *Citrobacter*, *Enterobacter*, *Enterococcus* and *Serratia* have been implicated in modulating pesticide resistance in various insect species, as reviewed in Thia et al. [45]. For example, gut bacteria like *Enterococcus* can both increase and decrease pesticide resistance in *P. xylostella* depending on the nature of the pesticide being applied [48]. Similarly, *Sphingomona*s has been reported to increase pesticide resistance in *Aphis gossypii* Glover [49]. An imbalance in gut microbiota can also incur negative effects on host fitness [50]. These findings suggest that gut microbes can affect pesticide resistance and fitness of their hosts. This highlights the importance of studying their communities in agriculturally important insect pests for potential applications in pest management strategies.

In Australia, information about the bacterial microbial infections in agriculturally important pests remains limited, although regional variation in the microbes of pest aphids has recently been characterized [51] as well as in parasitoids regulating pests [52]. For lepidopteran pests, *Wolbachia* infection has been documented in two closely related species: *P. xylostella*, where it occurs at low frequencies (1.5%) and its allied cryptic species *Plutella australiana* (Landry & Hebert) where it appears to be fixed [53]. The phenotypic effects of both these infections remain unknown. In addition, *Wolbachia* has also been found in local populations of *Ephestia kuehniella* (Zeller) where it causes partial cytoplasmic incompatibility and has no major fitness costs [54]. Lepidopteran pests in Australia include both invasive species like *S. frugiperda* and *P. xylostella* and native species like *Helicoverpa punctigera* (Wallengren), *Epiphyas postvittana* (Walker) and native armyworms (*Mythimna convecta*, *Persectania ewingii* and *Persectania dyscrita*). The presence of microbes locally (particularly endosymbionts) might be lower in invasive pests where bottlenecks have occurred during invasion, as suggested in aphids [55].

While there are a variety of ways of potentially using bacterial microbes for pest control strategies in Lepidoptera and other pests, an essential starting point is to identify which endosymbionts and gut microbes are present in pests locally. Without this baseline knowledge, it is difficult to assess the potential for bacteria to be used in transinfections to establish favourable traits or in other ways such as by informing chemical control methods [48]. The overall goal of this study was to characterize bacterial symbiont diversity and its distribution in agriculturally important lepidopteran pests found in Australia, with a focus on the state of Victoria. Our sampling prioritized lepidopterans from pasture, grain and vegetable crops, but we also included one species reared by a commercial biological control company that is used as a host for the commercial production of natural enemies. To achieve our research goal, this study focused on three main objectives. First, endosymbionts were broadly screened across several Lepidoptera species using specific primers via qPCR. Second, *Wolbachia* infections identified in some species were examined in greater detail to place them within a broader phylogenetic context. Third, selected candidate species were further analysed using 16S rRNA metabarcoding to comprehensively profile their microbiomes, with a particular focus on taxa potentially associated with pesticide resistance.

## 2. Materials and Methods

### 2.1. Lepidopteran Samples

#### 2.1.1. Sample Collection

**Figure 1:**
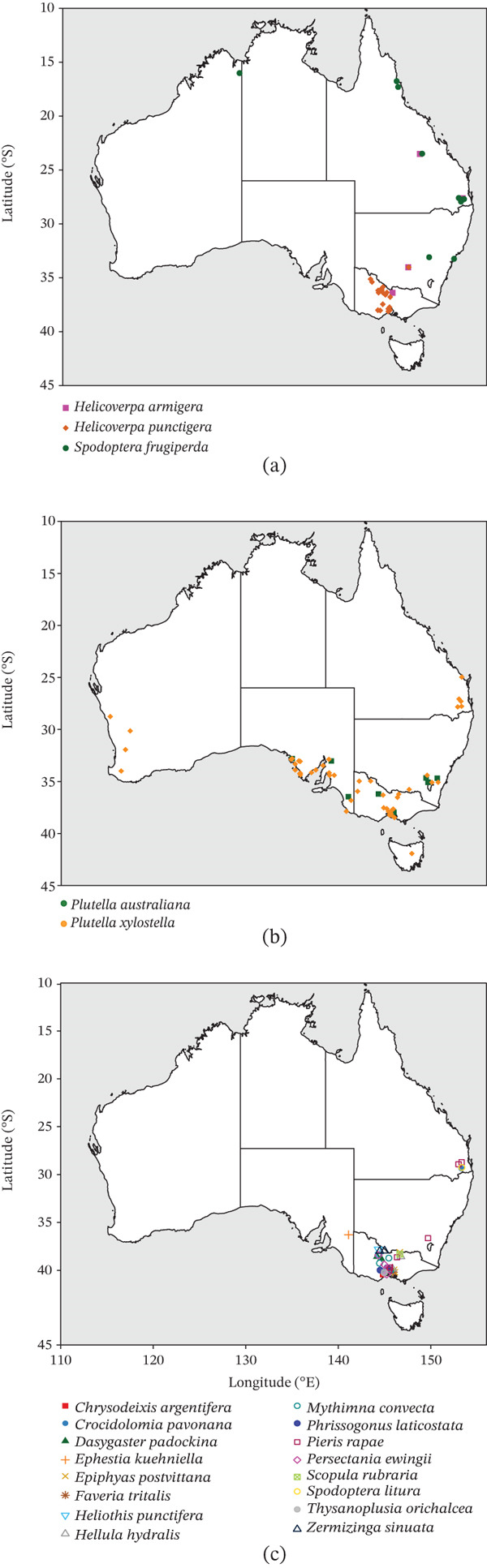
Sampling sites for field‐collected lepidopteran insect pests across Australia. Subfigures (a–b) show sites where target species were collected, whereas subfigure (c) shows sites for nontarget species sampling. Each data point represents a single population, defined as a set of samples of a species collected from one geographic site. In some cases, different species were collected at the same site, so their points may overlap.

Lepidopteran samples at different life stages (larvae, pupae and adults) were collected from 95 sites across Australia between 2021 and 2025 (Figure 1). Samples were primarily collected from commercial and volunteer grain, pasture and vegetable crops, and weeds along roadsides and fields, using direct searches and sweep‐netting. Opportunistic samples were also collected from fruit trees and vineyards. In most cases, live field‐collected lepidopterans were immediately stored in 100% ethanol. However, in some instances, larvae and pupae were brought to the laboratory at the Bio21 Institute (University of Melbourne, Melbourne, Victoria, Australia) for rearing for up to 3 weeks, which may have influenced gut microbial composition, and represent a potential confounding factor. Life stage was recorded for each specimen (Table 1) but was not included as a variable in downstream analyses due to uneven and limited sampling across stages. We specifically targeted *P. australiana*, *P. xylostella*, *S. frugiperda*, *H. armigera* and *H. punctigera* during sampling. Some nontarget species were also collected.

**Table 1:** List of lepidopteran species identified using CO1 barcoding and their endosymbiont infections.

Species	Host plants	No. of individuals/pooled extractions	No. of populations	States	Endosymbiont infection in this study	Percent infected (number infected)	Endosymbionts observed internationally	Life stage
*Crocidolomia pavonana*	Broccoli	2	1	QLD	—	—	—	Adult
*Chrysodeixis argentifera*	Lucerne	8	2	VIC	—	—	—	Mixed (pupa, adult)
*Dasygaster padockina*	Pasture	2	1	VIC	—	—	—	Adult
*Ephestia kuehniella*	Wheat flour	34	1	SA	*Wolbachia*	100% (34)	*Wolbachia*, Japan and United States ([56]; [57]; [58])	Mixed (larva, adult)
*Epiphyas postvittana*	Chilli, wattle, almond, European dewberry	12	4	VIC	*Wolbachia*	16.66% (2)	—	Mixed (larva, adult)
*Faveria tritalis*	Unknown	10	1	VIC	*Wolbachia*	100% (10)	—	Larvae
*Helicoverpa armigera*	Chickpeas, corn	53	4	NSW, QLD, VIC	*Spiroplasma*	7.55% (4)	None, Russia [59]	Mixed (larva, pupa, adult)
*Helicoverpa punctigera*	Cape weed, canola, chickpeas, perennial ryegrass, lucerne, red clover, strawberry clover, faba bean, common vetch	92	20	NSW, VIC	*Wolbachia*	1.08% (1)	—	Mixed (larva, pupa, adult)
*Helicoverpa punctigera*	Cape weed, canola, chickpeas, perennial ryegrass, lucerne, red clover, strawberry clover, faba bean, common vetch	92	20	NSW, VIC	*Rickettsia*	1.08% (1)	—	Mixed (larva, pupa, adult)
*Heliothis punctifera*	Canola, faba bean mix	2	2	VIC	—	—		Mixed (pupa, adult)
*Hellula hydralis*	Unknown	12	2	VIC	—	—		Mixed (larva, adult)
*Mythimna convecta*	Mustard, barley, hedge mustard, wheat, mixed pasture species and an unknown species	19	7	VIC	—	—		Mixed (larva, pupa, adult)
*Persectania ewingii*	Barley	3	3	VIC	—	—	—	Mixed (larva, pupa, adult)
*Phrissogonus laticostata*	Lucerne	2	2	VIC	—	—	—	Mixed (pupa, adult)
*Pieris rapae*	Canola, cabbage, forage brassica, wild radish, wild mustard	20	7	NSW, QLD, VIC	—	—	None [60]	Larva
*Plutella australiana*	Swinecress, sea rocket, wall rocket, wild brassica, forage brassica	43	9	ACT, NSW, SA, VIC	*Wolbachia*	100% (43)	—	Mixed (larva, adult)
*Plutella xylostella*	Broccoli, broccolini, choy sum, swinecress, canola, volunteer canola, sea rocket, wall rocket, chinese cabbage, wild radish, hedge mustard	288	48	QLD, SA, NSW, ACT, WA, VIC, TAS	*Wolbachia*	1.38% (4)	*Wolbachia*, Canada [61], United States [62], Malaysia, Kenya [63], Nepal ([64]), South America, North America, Africa, Asia [65]	Mixed (larva, adult)
*Scopula rubraria*	Grape vine	5	1	VIC	—	—	—	Larva
*Spodoptera frugiperda*	Corn	105	10	QLD, NSW, WA	—	—	*Arsenophonus* and *Spiroplasma*, Colombia ([66]; [67]), *Wolbachia*, China [68], Argentina, Puerto Rico, United States [69], None, France, United States, Zambia ([70]; [71])	Larva
*Spodoptera litura*	Broccoli	6	1	QLD	—	—	*Wolbachia,* India ([72]; [73])	Larva
*Thysanoplusia orichalcea*	Canola	1	1	VIC	—	—	—	Adult
*Zermizinga sinuata*	Lucerne	2	2	VIC	—	—	—	Adult

Abbreviations: ACT, Australian Capital Territory; NSW, New South Wales; QLD, Queensland; SA, South Australia; TAS, Tasmania; WA, Western Australia; VIC, Victoria.

*P. australiana*, *P. xylostella* and other nontarget Lepidoptera species larvae, particularly those attacking *Brassica* spp., were reared individually on cabbage leaves (*Brassica oleracea* var. *capitata*) in a microcosm (small, ventilated containers with mesh lids) at 19°C ± 1°C under a 16:8 h light:dark cycle. It is important to note that we were able to rear *P. australiana* for one generation on cabbage; however, we observed a low rate of oviposition and very slow larval growth. Perry et al. [53] reported no oviposition by this species on cabbage, confirming that it is a poor host. In contrast, *S. frugiperda* and *H. armigera* larvae were reared on an artificial diet described by Griffith and Smith [74] in glass vials at 25°C ± 1°C and a 16:8 h light:dark cycle until pupation. Evidence of parasitism in reared larvae was assessed daily. Once adults emerged, they were immediately stored in 100% ethanol at −20°C until the molecular work was performed.

We also included a subset of DNA extractions of *P. xylostella* (*n* = 155) and *P. australiana* (*n* = 28) samples collected in 2014–2016 as part of a previous study by Perry et al. [53]. The remaining samples of *P. xylostella* (*n* = 133) and *P. australiana* (*n* = 15) were collected in this study. In addition, *E. kuehniella* samples obtained from the commercial company, Biological Services (Loxton, South Australia, Australia), were included, which had been previously used in a study conducted by Zhao et al. [54].

#### 2.1.2. DNA Extraction Methods

DNA extractions were performed on individuals. We primarily used Chelex DNA extraction for samples reared in the laboratory for qPCR assay. The tissue used for DNA extraction consisted of either the whole body or a section of abdomen if the body size was too large. For Chelex DNA extraction, tissue was placed with 3 *μ*L of Proteinase K (20 mg/mL) along with 250 *μ*L of 5% Chelex‐100 (Bio‐Rad Laboratories, Gladesville, NSW) into 1.7‐mL Eppendorf tubes. Two 3‐mm stainless steel beads were added to homogenize the mixture using a TissueLyser II (Qiagen, Hilden, Germany) for 3 min at 30 Hertz. After homogenization, samples were centrifuged at maximum speed for 1 min and then incubated in a 65°C water bath for 1 h, followed by 10 min of incubation at 90°C water bath to inactivate Proteinase K and ensure complete tissue digestion.

We extracted high‐quality DNA for qPCR screening and 16S rRNA metabarcoding, using the DNeasy Blood and Tissue Kit (Cat. No. 69504) (Qiagen, Hilden, Germany), Roche High Pure PCR Template Preparation Kit (Germany) or the EZ2 Connect protocol (Cat. No. 9003210) (Qiagen, Hilden, Germany), following the manufacturer′s instructions. Extraction methods depended on their availability in the laboratory at the time of processing across the collection period. The use of multiple DNA extraction protocols may have introduced methodological variation and potential batch effects and should be considered when interpreting the results. We performed whole‐body extractions of individual insects, without separate dissection of the gut. In addition, surface sterilization was effectively achieved, as all insect samples were preserved in 100% ethanol. Our main focus was on primary lepidopteran pests, whereas other species were collected opportunistically. This meant an imbalance in sample sizes, and where these are particularly small (e.g., *Thysanoplusia orichalcea*, Table 1), inferences about bacterial composition should be treated as preliminary.

#### 2.1.3. COI Barcoding for Assigning Lepidoptera Taxonomy

COI barcoding was used to identify the taxonomy of Lepidopteran individuals. The universal arthropod primers HCO2198 (5 ^′^‐TAAACTTCAGGGTGACCAAAAAATCA‐3 ^′^) and LCO1490 (5 ^′^‐GGTCAACAAATCATAAAGATATTGG‐3 ^′^) were used to amplify a 658 base pair target COI fragment [75]. In total, 25 *μ*L of final volume was prepared for each PCR reaction, consisting of 1.25 *μ*L MgCl2 (50 mM, Bioline), 2.5 *μ*L of 10× Standard ThermoPol Reaction Buffer (B9015S; New England BioLabs, NEB), 2.0 *μ*L dNTPs (2.5 mM), 1.25 *μ*L of each forward primer and reverse primer (10 *μ*M), 0.4 *μ*L of IMMOLASE DNA polymerase (5 U/*μ*L, Bioline), 1.5 *μ*L of DNA template and 14.85 *μ*L of ddH₂O. Positive (known lepidopteran species) and negative (water) controls were included in each PCR assay. Thermocycling conditions followed those described in Qazi et al. [52]. PCR products were submitted for Sanger sequencing at Macrogen Inc. (Geumcheongu, Seoul, Republic of Korea). Sequencing was performed in a single reverse direction only for all samples. The resulting sequences were analysed and quality‐trimmed using Geneious Prime 2024.0.7 (https://www.geneious.com/) [76]. Species identification was done by performing BLAST searches [77] on the GenBank and Barcode of Life Data System (BOLD) web platforms, assigning taxonomy to the species level, or (if not possible) to the genus level.

### 2.2. Objective 1: Endosymbionts Screening in Lepidoptera Species

#### 2.2.1. Endosymbiont Screening With qPCR

For endosymbiont screening, a qPCR assay was used to detect nine different endosymbionts: *Arsenophonus*, *Cardinium*, *Hamiltonella*, *Regiella*, *Rickettsia, Ricketsiella*, *Serratia*, *Spiroplasma* and *Wolbachia*. We used these endosymbionts which are quite common in other insect species as a baseline for screening because there is limited information about the presence and diversity of endosymbiont infections in Lepidoptera worldwide; so far, only *Arsenophonus* and *Spiroplasma* [66, 67], *Rickettsia* [78] and *Wolbachia* [79] have been reported. The primer sequences used in this study for all targeted endosymbionts are the same as listed in Yang et al. [51]. DNA samples were initially diluted in a 1:3 ratio using water. If the sample was too concentrated after quantification through Qubit, it was further diluted to achieve a 1:10 ratio. We performed the qPCR assay using the 384‐well plate setup of Roche LightCycler 480 system (Roche Diagnostics Pty. Ltd) following the methodology described in Lee et al. [80]. A final volume of 10 *μ*L was prepared for each qPCR reaction mixture. Each reaction consist of 3.276 *μ*L of PCR‐graded water (Elga Purelab flex), 0.064 *μ*L of dNTP solution at 25 mM (Bioline), 1 *μ*L of the 10× ThermoPol reaction buffer (B9004S; New England BioLabs, NEB), 0.04 *μ*L of each forward and reverse primer at 100 *μ*M, 0.4 *μ*L of MgCl2 (50 mM, Bioline), 0.25 *μ*L of the LightCycler 480 High Resolution Melting Master (Roche), 0.01 *μ*L of IMMOLASE DNA polymerase (5 U/*μ*L, Bioline) and 2 *μ*L of diluted DNA. For each plate, positive (DNA from known endosymbiont) and negative (PCR‐graded water) controls were included to verify the accuracy of the DNA amplification, and to detect any contamination. The cycling conditions for the qPCR assay of *Arsenophonus*, *Cardinium*, *Hamiltonella*, *Regiella*, *Rickettsia*, *Rickettsiella*, *Serratia* and *Spiroplasma* were as described elsewhere [52], except that the annealing temperatures differed for two endosymbionts: 65°C for *Rickettsiella* and 61°C for *Serratia.* We used a TaqMan TAMRA probe assay for the *Wolbachia* assay [52], following the same cycling conditions and annealing temperature described in our previous study.

Conventional PCR (cPCR) sequencing was performed for further validation of endosymbiont infection in the samples, particularly those with qPCR *C*_P_ values between 30 and 35. For a few samples, only PCR and 16S rRNA metabarcoding were performed without qPCR; this occurred if the endosymbiont density was under the detection limit in qPCR assays. We prepared a final volume of 25 *μ*L for each PCR reaction mixture. Each reaction contained 14.85 *μ*L of PCR‐graded water (Elga Purelab flex), 1.25 *μ*L of each forward primer and reverse primer (10 *μ*M), 1.25‐*μ*L MgCl_2_ (50 mM, Bioline), 2.5 *μ*L of 10× Standard ThermoPol reaction buffer (B9015S; New England BioLabs, NEB), 2.0‐*μ*L dNTPs (2.5 mM), 0.4 *μ*L of IMMOLASE DNA polymerase (5 U/*μ*L, Bioline) and 1.5 *μ*L of DNA template. For each run, positive and negative controls were included. The cycling conditions for PCR are as follows: initial denaturation at 95°C for 10 min, followed by 45 cycles of denaturation at 94°C for 1 min, annealing at 55°C for 1 min, extension at 72°C for 1 min and a final extension at 72°C for 7 min. A 5 *μ*L of final PCR product was then loaded on 2% agarose gel stained with Syber Safe DNA Gel Stain (Invitrogen) for the visualization of DNA bands.

### 2.3. Objective 2: Phylogenetic Analysis of *Wolbachia*

We found that *Wolbachia* was the most predominant endosymbiont in our study (see Results).

We used multilocus sequence typing (MLST) to characterize *Wolbachia* strains for reliable strain classification, as recombination in *Wolbachia* can mislead single‐gene phylogenies and may facilitate the horizontal spread of *Wolbachia*‐induced reproductive host phenotypes between strains [81]. Therefore, we performed the MLST using the *coxA*, *gatB*, *hcpA*, *ftsZ* and *fbpA* genes following the primer sets described by Baldo et al. [82] and amplified the *wsp* genes using primers from Kondo et al. [83]. For PCR cycling conditions and agarose gel visualization, we followed the protocols described by Richardson et al. [84]. PCR products were then Sanger sequenced in both directions (Macrogen Inc., Geumcheongu, Seoul, Republic of Korea). Forward and reverse sequences were manually assessed for quality and edited using Geneious Prime 2024.0.7 (https://www.geneious.com). Each gene sequence was compared against PubMLST database to identify matches with previously reported MLST alleles [85], which were then used to generate allelic profiles for each sample. The concatenated multigene sequences of each sample further supported identification of matches to previously reported sequence types (STs) in the PubMLST database. *Wolbachia* MLST locus sequences obtained in this study were assembled in Geneious Prime 2024.0.7 and subsequently aligned using MUSCLE in MEGA 11 [86], together with sequences from *Wolbachia* strains representing different supergroups available in the PubMLST public database [87].

For Supergroup A, the following reference species were used: *Aedes albopictus* (Skuse), *Diadegma semiclausum* (Hellen), *Drosophila simulans* (Sturtevant), *E. kuehniella*, *Hypolimnas bolina* (Linnaeus), *Nasonia longicornis* (Darling) and *Nasonia vitripennis* (Walker). For Supergroup B, we used the following reference species: *E. kuehniella*, *Eurema mandarina* (De L′Orza), *Eurema hecabe* (Linnaeus), *Lycaeides idas* (Linnaeus), *Ostrinia scapulalis* (Walker) and *Spodoptera exempta* (Walker). We used *Brugia malayi* (Brug) (Supergroup D) and *Cimex lectularius* (Linnaeus) (Supergroup F) as an outgroup. We used the MLST and *wsp* gene for *D. semiclausum* from our previous study [52]. Further, we obtained the *wsp* gene sequence for *S. exempta* from GenBank (Accession number: JN656949.1) due to the unavailability of *wsp* gene in PubMLST. The *Wolbachia* strain in commercial *E. kuehniella* was previously confirmed by Zhao et al. [54] using the *ftsZ* and *wsp* genes. However, to obtain a complete allelic profile, we performed MLST analysis across all loci.

In this study, we generated three phylogenetic trees to represent different gene combinations and to address variation in gene sampling across species. For each tree, the best fit nucleotide substitution model was selected in MEGA11 using the Bayesian Information Criterion (BIC). Phylogenetic trees were then constructed in MEGA11 using the maximum likelihood method with 1000 bootstrap replicates. For all three phylogenetic trees, we used the Tamura‐3 parameter model (T92 + G) [88].

The first phylogenetic tree, based on all five MLST genes, included all species except *H. punctigera*, for which the complete MLST set could not be amplified. The second phylogenetic tree included one MLST gene *fbpA*, which was successfully amplified in *H. punctigera*, allowing inclusion of all species. The third phylogenetic tree was based only on the *wsp* gene, a hypervariable locus that evolves more rapidly than most other *Wolbachia* genes [89, 90].

### 2.4. Objective 3: Microbiomes Across Lepidoptera Species

#### 2.4.1. Microbiome Screening With 16S rRNA Metabarcoding

**Figure 2:**
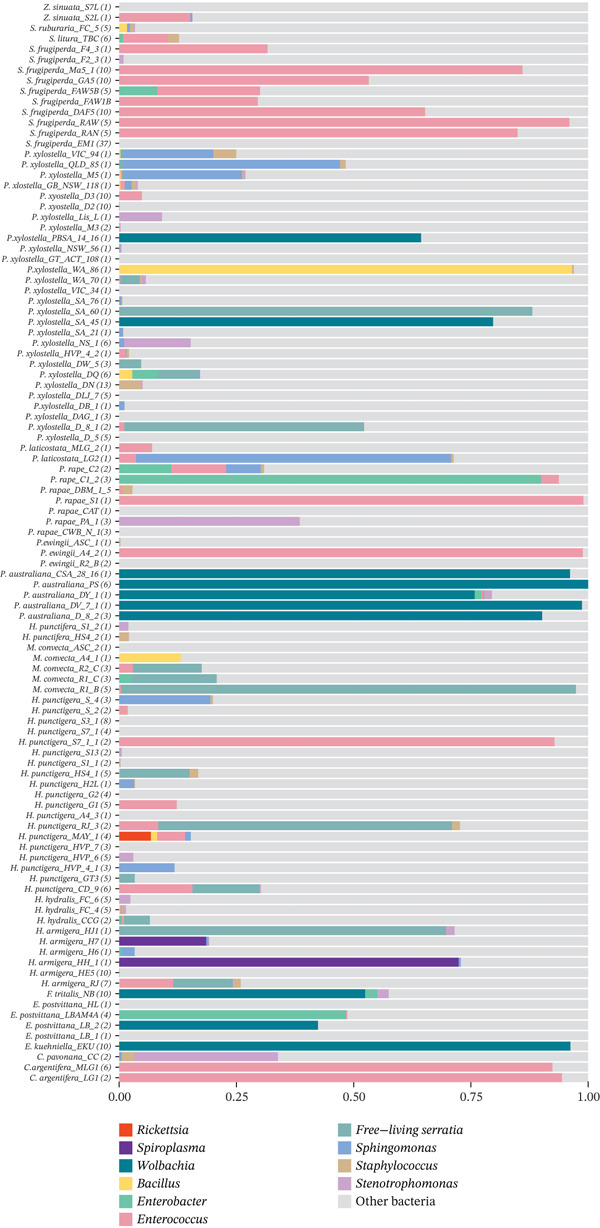
Relative abundance of bacterial taxa detected in lepidopteran samples using 16S rRNA metabarcoding. The *x*‐axis represents the mean relative abundance of Illumina reads per sample, whereas the y‐axis lists each sample, including species name, sample ID and the total number of individuals per pool or sample (in parentheses). Bacterial taxa are colour‐coded as indicated in the legend.

We more broadly screened the microbiome of sampled lepidopterans using 16S rRNA metabarcoding, which also provided validation of the infections observed using qPCR. We performed 16S rRNA metabarcoding for all species except *Dasygaster padockina* (Le Guillou), which had only Chelex‐extracted DNA with no additional samples available, and *T. orichalcea* (Fabricius), which had insufficient DNA for metabarcoding. The number of biological replicates per lepidopteran species is provided in the second column of Table 2, whereas the number of individuals within each biological replicate is shown on the *y*‐axis of Figure 2 (in parentheses). We focused on seven candidate bacterial genera previously associated with pesticide resistance in the gut microbiota of various insect pests: *Bacillus*, *Enterobacter*, *Enterococcus*, free‐living *Serratia*, *Sphingomonas*, *Staphylococcus* and *Stenotrophomonas* [45]. All remaining bacteria were grouped as ‘other bacteria’. High‐quality DNA was extracted from different life stages (see Table 1) and submitted to Novogene (Novogene Co. Ltd, Hong Kong) for library construction using the universal 341F and 806R primers [91]. The remaining 16S rRNA metabarcoding data were analysed using the protocol described in Yang et al. [51]. A total of 18,645,871 reads (mean = 152,835 reads per sample) were retained after each of the quality filtering and assembly steps.

**Table 2:** Detection of *Enterococcus* in lepidopteran hosts using 16S rRNA metabarcoding. Data are presented as positive samples/total tested, with read counts shown for *Enterococcus* and *E. mundtii*. Samples with > 1000 reads were selected for analysis, although a few samples with fewer reads were included when only limited data were available.

Host species	Proportion of samples containing *Enterococcus* (samples Enterococcus detected/total number of 16S‐sequenced samples)	Total *Enterococcus* ASV reads	Total *Enterococcus mundtii* ASV reads	Percent *Enterococcus mundtii* of *Enterococcus*
*Chrysodeixis argentifera*	2/2	442,577	351,474	79.41
*Epiphyas postvittana*	1/4	346	0	0
*Helicoverpa armigera*	1/6	39,464	0	0
*Helicoverpa punctigera*	7/18	196,892	21,473	10.90
*Hellula hydralis*	2/3	968	0	0
*Mythimna convecta*	2/5	2249	2249	100
*Phrissogonus laticostata*	2/2	11,449	9480	82.80
*Pieris rapae*	3/6	188,011	188,011	100
*Plutella australiana*	1/4	615	0	0
*Plutella xylostella*	4/29	11,921	9763	81.89
*Persectania ewingii*	1/3	174,376	0	0
*Spodoptera frugiperda*	8/10	644,941	479,042	74.27
*Spodoptera litura*	1/1	14,571	0	0
*Zermizinga sinuata*	1/2	24,429	0	0

#### 2.4.2. Microbial Community Composition Using Robust Aitchison PCA

We assessed differences in microbiome composition across samples using Robust Aitchison principal component analysis (PCA) implemented in DEICODE within QIIME 2 described by Martino et al. [92]. This approach is robust to sparsity and compositionality in microbiome data [93]. Pairwise distances between samples were calculated in the Aitchison‐transformed space, and these distances were visualized in Emperor. A minimum feature count of 200 threshold was applied to retain samples with sufficient sequencing depth for robust analysis. This approach allowed us to visualize differences in microbial composition between samples, but it does not represent classical beta diversity since it is based on transformed feature data rather than traditional abundance‐based diversity metrics.

Furthermore, for a subset of taxa, we tested for differences among species and/or the influence of host crops and geographic locations, using a Permutational Multivariate Analysis of Variance (PERMANOVA) with 999 permutations. Pairwise PERMANOVA *p* values were adjusted using false discovery rate (FDR) correction to account for multiple comparisons, and adjusted *q*‐values are all larger than 0.05. We conducted this analysis on *P. xylostella* as we had a sufficient number of samples to compare microbiome communities among populations collected from different Australian states and host plants. In addition, we had an adequate number of *Helicoverpa* samples, which allowed us to compare the microbiomes of *H. punctigera* and *H. armigera*, as well as to examine variation among samples collected from different host plants. However, the *H. punctigera* samples were only collected from Victoria, which limited our ability to perform location‐based analyses for this species.

We estimated alpha diversity at the family level to provide a consistent and comparable taxonomic unit across samples [94]. For each species, alpha diversity was expressed as observed bacterial family richness relative to the maximum observed within that species (observed families divided by the species‐specific maximum). This normalized metric was used to facilitate comparison of richness variation among individuals within species. Values were visualized as boxplots to complement the beta diversity patterns shown in the PCA.

## 3. Results

### 3.1. Objective 1: Endosymbionts Screening in Lepidoptera Species

#### 3.1.1. Species Identification and Endosymbiont Screening

We identified 21 lepidopteran pest species collected from 95 sites across Australia between 2021 and 2025 using COI barcoding (Table 1). Of these, 15 species had no endosymbiont infection (Table 1). The most abundant endosymbiont detected was *Wolbachia*, which was found in six species (Table 1). *Wolbachia* exhibited polymorphic infections among and within populations of some species (Table 1).

Among the 20 populations of *H. punctigera* screened, only one population had a single *Wolbachi*a‐positive individual (1/5), whereas the remaining 19 populations showed no infection. However, we were unable to detect *Wolbachia* in the putative *Wolbachia*‐positive *H. punctigera* sample using 16S rRNA metabarcoding, and we consider the detection inconclusive. In this study, detections are considered conclusive only when confirmed across the methods used. In *P. xylostella*, out of the 48 populations screened, four populations had a single *Wolbachia*‐positive individual, all from samples collected by Perry et al. [53] in 2014–2016. For *E. postvittana*, two individuals sampled from a single population were infected with *Wolbachia*. In *P. australiana*, we confirmed 100% *Wolbachia* infection from nine populations screened, as reported previously [53]. In commercial *E. kuekniella, Wolbachia* infection was at 100% in all individuals screened as reported previously [54]. Other endosymbionts found were *Spiroplasma* in a small number of individuals (4/9) in a single population of *H. armigera*, and a single infection of *Rickettsia* in a population of *H. punctigera* (1/4). The results of endosymbiont screening are briefly summarized in Table 3.

**Table 3:** Summary of *Wolbachia* (Wb), *Rickettsia* (Rc) and *Spiroplasma* (Sp) infections in field‐collected and commercial lepidopteran insect pests using conventional PCR (cPCR), quantitative PCR (qPCR) and 16S rRNA metabarcoding (16S).

Family	Species	Source	Wb (qPCR)	Wb (cPCR)	Wb (16S)	Rc (qPCR)	Rc (cPCR)	Rc (16S)	Sp (qPCR)	Sp (cPCR)	Sp (16S)
Pyralidae	*Ephestia kuehniella*	Commercial	×	×	×						
Tortricidae	*Epiphyas postvittana*	Field	×	×	×						
Pyralidae	*Faveria tritalis*		×	×	×						
Noctuidae	*Helicoverpa armigera*								×	×	×
Noctuidae	*Helicoverpa punctigera*		×	×		×	×	×			
Plutellidae	*Plutella australiana*		×	×	×						
Plutellidae	*Plutella xylostella*		×	×	×						

### 3.2. Objective 2: Phylogenetic Analysis of *Wolbachia*

**Figure 3:**
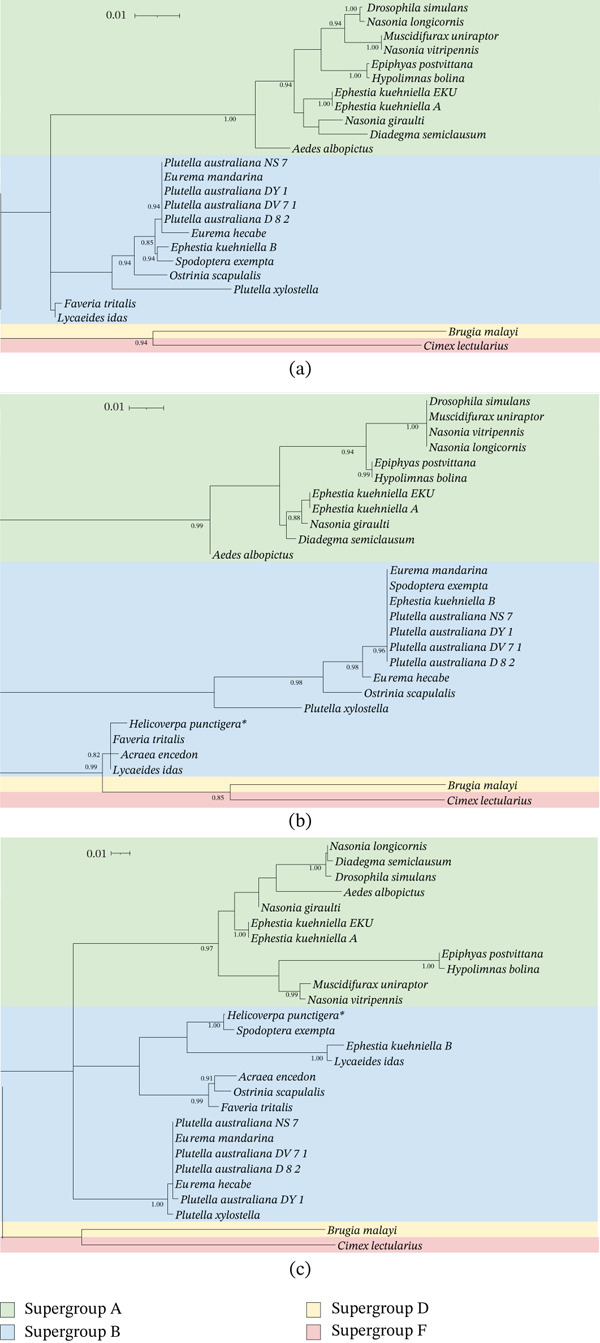
Maximum likelihood phylogenetic trees constructed for *Wolbachia* MLST and *wsp* genes from lepidopteran insect pests (highlighted in bold), alongside known *Wolbachia* strains from other insects obtained from the PubMLST database. Branch lengths represent the number of nucleotide substitutions (refer to the scale at the top center), and node labels indicate bootstrap support values greater than 0.80. Samples from this study are shown in bold. The shading corresponds to *Wolbachia* supergroups (see legend at the top left). (a) Phylogenetic tree constructed from five MLST genes, excluding *Helicoverpa punctigera*. (b) Phylogenetic tree constructed from one MLST genes (fbpA), including *Helicoverpa punctigera*. (c) Phylogenetic tree constructed from the *wsp* gene, including *Helicoverpa punctigera.* ∗ *Wolbachia* detection is inconclusive.

Maximum likelihood phylogenetic trees, based on (i) five MLST genes excluding *H*. *punctigera*, (ii) one MLST gene including *H*. *punctigera* and (iii) *wsp* genes from all species, are shown in Figure 3a–c, respectively. The phylogenetic tree of the five MLST genes (Figure 3a) indicated that *Wolbachia* detected in *E. postvittana* and commercial *E. kuehniella* belong to Supergroup A with the latter finding already reported in our previous study [54]. *Wolbachia* detected in all *P. australiana* individuals, *P. xylostella* and *F. tritalis* belong to Supergroup B. Furthermore, our phylogenetic tree of one MLST gene based on *fbpA* (Figure 3b) and *wsp* (Figure 3c) identified the *Wolbachia* strain in *H. punctigera* also belonging to Supergroup B, but this assignment requires further work given that not all MLST genes were amplified for this species and given that we failed to detect *Wolbachia* based on the 16S rRNA metabarcoding (Table 3). All phylogenetic trees showed consistent supergroup assignments, although the branching patterns within each supergroup clade varied (Figure 3). The differences observed among the three trees likely reflect minor variations in the evolutionary signals captured by different genes [95, 96]. The *wsp* gene evolves more rapidly and may be subject to different selective pressures compared with the MLST genes [97].

We compared the MLST profiles of *Wolbachia* from our lepidopteran samples with those in the PubMLST database to determine their STs. The *E. kuehniella* sample matched ST‐92, and all individual samples of *P. australiana* matched ST‐41. All remaining *Wolbachia* isolates obtained in this study exhibited novel MLST profiles and therefore represent new STs (Table 4).

**Table 4:** MLST allelic profiles of *Wolbachia* from field‐collected lepidopteran species and the commercial *Ephestia kuehniella*. Supergroup assignments are based on results from our phylogenetic analysis. For each gene, the closest matching allele in PubMLST is provided, with the number of nucleotide differences shown in parentheses where applicable. Sequence type refers to exact multigene sequence matches recorded in PubMLST.

Species	Supergroup	*coxA*	*gatB*	*hcpA*	*ftsZ*	*fbpA*	Sequence type (ST)
*Epiphyas postvittana*	A	6	7	7(1)	3	8	^b^
*Ephestia kuehniella*	A	59	54	68	3	67	ST‐92
*Plutella australiana* (D_8_2)	B	14	39	40	36	4	ST‐41
*Plutella australiana* (DV_7_1)	B	14	39	40	36	4	ST‐41
*Plutella australiana* (DY_1)	B	14	39	40	36	4	ST‐41
*Plutella australiana* (NS_7)	B	14	39	40	36	4	ST‐41
*Plutella xylostella*	B	4(11)	39	40(1)	7(1)	162(18)	^b^
*Faveria tritalis*	B	14	39	119	7(25)	9	^b^
*Helicoverpa punctigera*	B	^a^	^a^	^a^	^a^	198	^b^

^a^ *Helicoverpa punctigera* lacks *coxA*, *gatB*, *ftsZ and hcpA* genes, and assignment is therefore tentative.

^b^No exact matches in PubMLST.

### 3.3. Objective 3: Microbiomes Across Lepidoptera Species

#### 3.3.1. 16S rRNA Metabarcoding of Microbiome

The 16S rRNA metabarcoding analysis identified a diverse and abundant microbiome across the characterized Lepidoptera: *Bacillus*, *Enterobacter*, *Enterococcus*, free‐living *Serratia*, *Sphingomonas*, *Staphylococcus* and *Stenotrophomonas*. All remaining bacteria were grouped as ‘other bacteria’. Good′s coverage values were calculated for all 103 samples using QIIME2 to assess sequencing depth and sampling completeness. All samples achieved coverage values greater than 0.996, with the majority at or very close to 1.0 (mean = 0.9998). These results indicate that the sequencing depth was sufficient to capture the microbial diversity present across all samples. The targeted bacteria were detected at varying relative abundances across the sampled species. *Enterococcus* was the most frequently detected and found in 14 species. The other six bacteria (Figure 2), such as *Bacillus*, free‐living *Serratia*, *Stenotrophomonas*, *Enterobacter*, *Sphingomonas* and *Staphylococcus*, were detected in five, five, nine, 10, 10 and 12 species, respectively. Since our 16S rRNA metabarcoding results did not assign some bacteria down to the species level, we manually confirmed all ASVs using BLAST searches in the NCBI database. We found the highest number of *Enterococcus* ASV reads in *S. frugiperda,* approximately 644,941 reads. Among the *Enterococcus* reads, the *Enterococcus mundtii* related ASV accounted for around 74.28% of these reads with sequence identity greater than 99.8%, whereas other *Enterococcus* species comprised 25.72% of the reads. *Enterococcus*, including *E. mundtii*, was also detected in several other species, showing notable differences in abundance compared with *S. frugiperda* (see Table 2).

*Serratia* ASV reads were highly abundant across our samples compared with other target bacterial taxa. BLAST results of *Serratia* ASV counts indicated that both *H. armigera* and *P. xylostella* were infected with *Serratia marcescens*, with PCR confirmation obtained for *H. armigera*. *Serratia fonticola* was detected in *H. punctigera* and *P. ewingii*, whereas multiple other *Serratia* species were detected in *M. convecta*.

#### 3.3.2. Microbial Community Composition Using Robust Aitchison PCA

**Figure 4:**
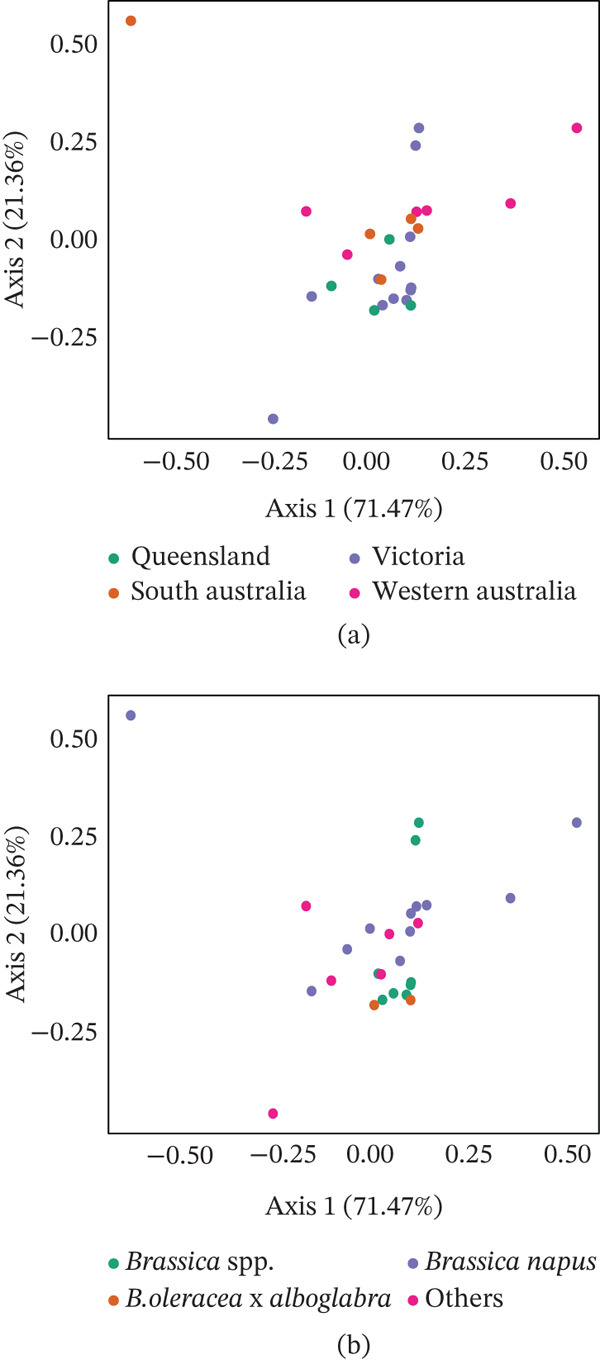
Robust Aitchison PCA (RPCA) in bacterial communities calculated using DEICODE. (a) Variation among *P. xylostella* samples across geographic locations. (b) Variation among *P. xylostella* samples across different crop hosts. Other crops (*Brassica oleracea* var. *italica*, *Brassica rapa* subsp. *parachinensis*, *Brassica rapa* subsp. *pekinensis*, *Lepidium coronopus*, *Cakile maritima* and *Cucurbita* spp.) were grouped as ‘Others’ due to limited sample sizes for PERMANOVA.

**Figure 5:**
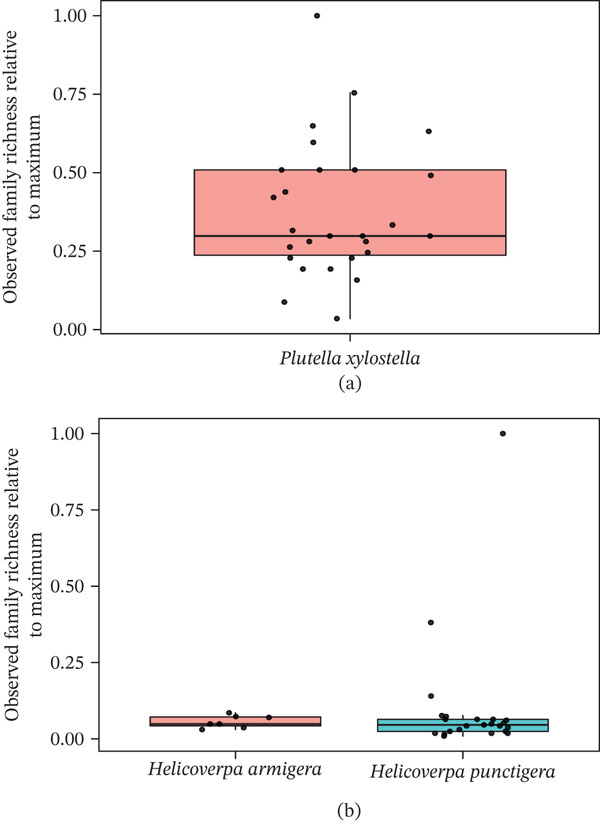
Relative richness of bacterial families in Lepidoptera species. (a) *Plutella xylostella.* (b) *Helicoverpa armigera* and *H. punctigera* samples. Boxplots with individual sample points overlaid show the distribution of observed bacterial families relative to the maximum.

**Figure 6:**
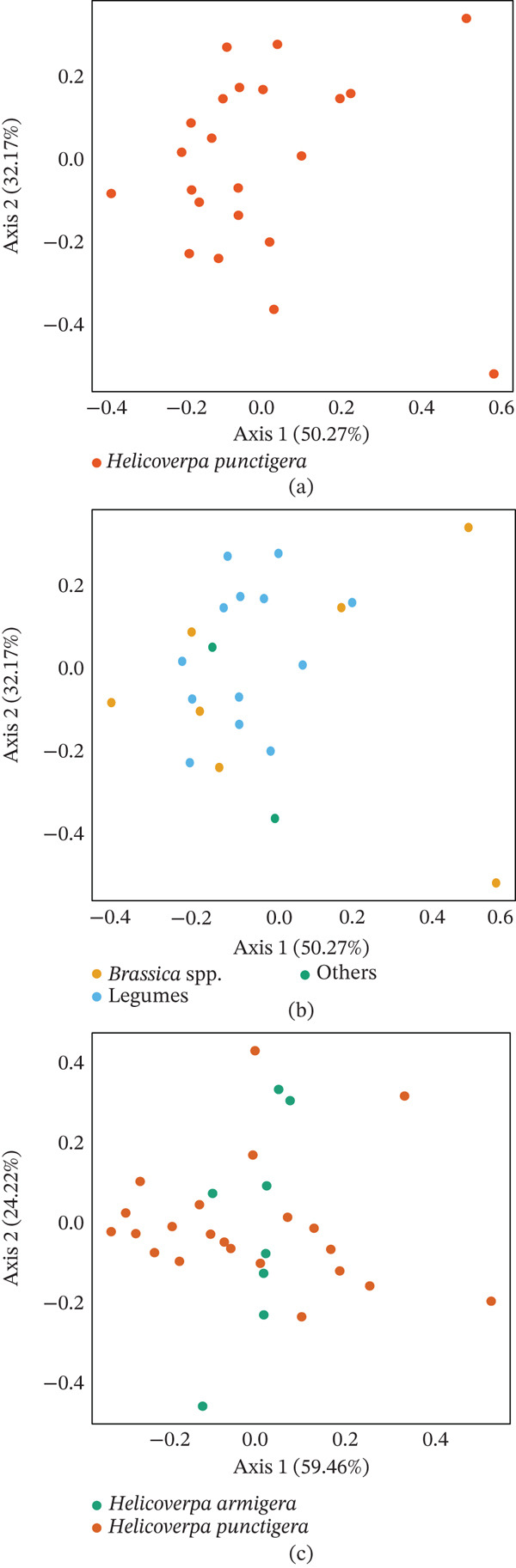
Robust Aitchison PCA (RPCA) of beta diversity in bacterial communities calculated using DEICODE. (a) *Helicoverpa punctigera* samples collected from Victoria. (b) Variation among *H. punctigera* samples across different crop hosts, grouped into three categories: (i) *Brassica* spp. (*Brassica napus*, *Brassica* sp.), (ii) Legumes (*Vicia sativa*, *Cicer arientinum*, *Trifolium fragiferum*, *Medicago sativa*, *Cicer arientinum* and *Vicia faba* (mixed legumes), *Trifolium* and *Vicia* spp. (mixed pasture), *Trifolium pratense*) and (iii) Others (*Arctotheca calendula*, *Sisymbrium officinale*/grassland). (c) Comparison between *Helicoverpa armigera* (green) and *H. punctigera* (orange).

For *P. xylostella* samples (Figure 4a,b), the first two axes in PCA explained 71.47% and 21.36% of the total variation, respectively. The compositional PCA analyses showed no significant differences in microbial community composition. Alpha diversity, measured as the number of bacterial families relative to the maximum observed in the species [94], was low to moderate and varied across individuals, indicating that while the overall composition is similar, individual samples differ in the number of bacterial families they host (Figure 5a). Based on PERMANOVA, the effects of location (*p* = 0.22) and crop host (*p* = 0.23) were statistically nonsignificant, suggesting that geography and host crop did not influence microbial community structure in *P*. *xylostella*. Similarly, PCA for *H. punctigera* (Figure 6a,b) captured 50.27% and 32.17% of the variation on the first two axes. No significant differences were observed among crop hosts (PERMANOVA, *p* = 0.64).

Finally, comparison between *H. armigera* and *H. punctigera* (Figure 6c) showed that the first two axes explained 59.46% and 24.22% of the variation (83.68% total). PERMANOVA indicated no significant differences between species (*p* = 0.92). Consistent with this, observed bacterial family richness relative to the species‐specific maximum was generally similar across *H. armigera* and *H. punctigera* samples (Figure 5b). Most samples fell within a comparable range, with the exception of two *H. punctigera* samples that were outliers. Together, these results indicate that both species host similar levels of bacterial family diversity and that differences in community composition are not driven by differences in alpha diversity.

## 4. Discussion

### 4.1. Limited Endosymbiont Diversity in Lepidopteran Species

Our screening of common insect endosymbionts revealed very limited diversity in the 22 lepidopteran species sampled from Australian agricultural systems. The most common infection identified in our study was *Wolbachia*, which were fixed in some species (*E. kuehniella*, *P. australiana*, *F. tritalis*) and exhibiting polymorphism within and among populations in others (*E. postvittana*, *H. punctigera*, *P. xylostella*) (Table 1). We also document the first potential case of polymorphic/rare *Spiroplasma* infection in *H. armigera*, a major agricultural pest worldwide, known for its extensive host range and broad geographic distribution [11, 13, 98]. *Spiroplasma* is known for its male‐killing reproductive effects in some species including Lepidoptera [99–101], so future assessment in *H. armigera* is of interest.

Our findings place *Wolbachia* frequency at the lower end of previous surveys of endosymbionts in Lepidoptera with *Wolbachia* often present in up to 80% of species [102]. Other endosymbionts including *Spiroplasma* were also detected in a number of species, as reviewed in Duplouy and Hornett [26]. We also note a wide range of infection frequencies across and within populations, which may relate to several factors including the nature of the phenotypes influenced by the endosymbionts in Lepidoptera [26] as well as climatic factors [79]. The relatively low incidence in our study may reflect the targeted nature of the survey and in some cases the relatively low number of populations and individuals sampled.

### 4.2. *Wolbachia* Supergroups A and B Dominated Among Lepidopteran Species

We found that *Wolbachia* was the most prevalent endosymbiont in Lepidoptera consistent with other studies [26]. In *P. xylostella*, we also confirmed rare cases of *Wolbachia* infection in samples derived from Perry et al. [53] collected between 2014 and 2016, although none of our own samples from 2021 to 2024 tested positive. Our phylogenetic analysis indicated that the *Wolbachia* strain in *P. xylostella* belongs to Supergroup B and is most similar to plutWB1 [63]. This is distinct from the strain reported by Perry et al. [53], which, based on the *wsp* gene, belonged to Supergroup A and was identical to plutWA1 [63]. Note that we performed MLST on only one of the four *Wolbachia*‐positive *P. xylostella* samples from Perry et al. [53], an individual that was not among the subset of *Wolbachia*‐positive *P. xylostella* (*n* = 5) previously sequenced in their study. The occurrence of two different *Wolbachia* strains in separate Australian *P. xylostella* contrasts with a US study which found both A and B strains in the same individual [62] but is consistent with other reports of single A‐ or B‐strain infections in this species [61, 63, 64]. Delgado and Cook [63] screened *P. xylostella* from 10 different countries and identified *Wolbachia* infections in samples from three countries, with three distinct strains, suggesting a geographically limited and polymorphic distribution. One strain (plutWB1) was linked to female‐biased sex ratios, though the infection rate was the same in both sexes [63]. A later global survey [65] detected a similar strain (plutWB1) at low prevalence (7%) across six continents, suggesting possible horizontal transfer from butterfly host species [79].

We also confirmed a similar widespread *Wolbachia* infection in the *P. australiana* samples sourced from a study by Perry et al. [53], which were collected between 2014 and 2016, and in our own collections from 2021 to 2024. Perry et al. [53] observed no sex‐ratio distortion in *Wolbachia*‐infected *P. australiana.* However, near fixation of a *Wolbachia* infection, coupled with a discordance between low mtDNA diversity and high nuclear diversity, can imply that a *Wolbachia* infection recently spreading through reproductive incompatibility has driven its linked mtDNA haplotypes through a population [103]. This may have occurred in the Australian range of *P. australiana* [53]. Curing experiments could illuminate the potential phenotypic effects of *Wolbachia* in this species. Our phylogenetic analysis shows that the *Wolbachia* strain in *P. australiana* belongs to Supergroup B, as previously reported [104]. We found that *Wolbachia* strain was identical to a strain in *E. mandarina*, where single‐strain infections induce cytoplasmic incompatibility, whereas double infections with two distinct strains induce feminization [30, 105, 106]. Similarly, Ward and Baxter [104], who first reported the genome of *Wolbachia* in *P. australiana*, *w*Aus, found that it was most similar to *w*Pip, the strain found in *Culex quinquefasciatus* based on MLST genes. Strain *w*Pip is known to cause cytoplasmic incompatibility [107–109] through two genes, *cifA* and *cifB* [110], though these have not yet been identified from the *w*Aus strain [104].

Our study reports the first documented case of *Wolbachia* and *Rickettsia* in *H. punctigera.* Despite the wide distribution of *Rickettsia*, so far only *Rickettsia felis* has been reported in Lepidoptera [78]. Our *Rickettsia* sp. strain in *H. punctigera* is identical to those found in whiteflies, where they confer fitness benefits [111–113] and improve host efficiency in virus transmission [114], though rarely impose any fitness cost [115]. Although 16S rRNA metabarcoding did not detect *Wolbachia* in *H. punctigera*, likely due to pool amplification caused by mismatch of the generic 16S V3‐4 primer, its presence was confirmed by PCR and qPCR. The COI sequences matched perfectly, ruling out contamination or parasitism, and BLAST of *wsp* and *fbpA* showed similarity to *Wolbachia* from other Lepidoptera species. We were unable to amplify the remaining MLST genes, likely due to low infection levels, but the available data support that this is most likely genuine, low‐titer *Wolbachia* infection.

We report the first documented case of *Wolbachia* infection in *E. postvittana* although in only one out of three populations. *E. postvittana* is a leaf‐rolling pest, native to Australia, that affects a wide range of horticultural crops [116, 117]. Our MLST and *wsp* phylogenetic trees show that the *Wolbachia* strain in *E. postvittana* is closely related to the strain from *H. bolina*, where it is known to cause male killing [118, 119]. Previous work by Geier et al. [120] in Australia have suggested that some populations of *E. postvittana* contain females with a male‐killing phenotype that produces approximately half the number of eggs produced by females without this phenotype. Given the reported sex‐ratio distortion and *Wolbachia* strain identity with a strain causing male‐killing in another host, it would be worthwhile exploring this further. Such phenotypic effects in *E. postvittana* may offer insights for managing this local pest.

### 4.3. Diverse Microbiomes Across Lepidopteran Species

In contrast to endosymbionts, the broader microbiomes of sampled lepidopteran species were more diverse. We focused our analysis on *Bacillus*, *Enterobacter*, *Enterococcus*, free‐living *Serratia*, *Sphingomonas*, *Staphylococcus* and *Stenotrophomonas* because these have previously been linked to pesticide resistance, reviewed in Thia et al. [45]. Of these candidate species, *E. mundtii* was the most dominant species detected, found in *S. frugiperda* particularly (see Table 3). *E. mundtii* and other *Enterococcus* species have been linked to positive effects on host physiology, fitness and pesticide resistance [50, 121–123]. Work in Chinese populations of *P. xylostella* has also indicated a complex role of *Enterococcus* species in both increasing and decreasing resistance to different pesticides [48, 124, 125]. However, in our survey, we did not detect *Enterococcus* often in Australian *P. xylostella*, but we did find *Sphingomonas,* which has been linked to pesticide resistance in aphids [49]. This may be an interesting area for future research, given the range of pesticide resistances found in Australian populations of *P. xylostella.*

In addition, we also detected *S. marcescens* in a few samples of *P. xylostella* and *H. armigera*. *S. marcescens* has been widely recognized for its entomopathogenic properties, affecting various insect species [126]. Several studies have documented its pathogenicity against agricultural pests including *H. armigera* and *P. xylostella* [126, 127]. Interestingly, Xia et al. [124] also reported that *Serratia* sp. found in the gut of *P. xylostella* increased its susceptibility to insecticides. However, *Serratia* can also provide beneficial effects. For example, in the planthopper, *Nilaparvata lugens* (Stål), *Serratia* can confer resistance to pesticides [128]. In our study, we cannot confirm whether *S. marcescens* was present throughout the insect body or restricted to the gut, since we performed whole‐body extractions rather than analysing the gut specifically. Future studies in identifying and characterizing the role of *Serratia* could help assess its potential as a biocontrol agent in key lepidopteran pests.

Our microbial compositional analysis (which involved whole body analysis rather than focusing on the gut tissue) showed no significant effect of geographic location or host plant on field‐collected *P. xylostella* and *H. punctigera*, microbiomes or even a difference between the related species *H. armigera* and *H. punctigera*. These findings are consistent with previous reports that laboratory‐reared different *P. xylostella* populations maintain relatively stable microbiomes [64] but contrast with some previous studies in China, India and United States that reported populations of *Spodoptera exigua* (Hübner) and *P. xylostella* with distinct microbial communities depending on geographic location and host crop [129–132]. The reason for these inconsistent findings is unclear. They may reflect differences in the ways that beta diversity is being evaluated. In our case, we used Aitchison distance in our comparisons, which is recommended for proportional comparisons of biodiversity [93]. In contrast, other studies often use Bray–Curtis distances that can lead to differences in conclusions about levels of biodiversity across treatments especially when using compositional datasets [133, 134]. We also did not include *P. australiana* in comparison analysis with its sibling species *P. xylostella* because in this species the large number of sequence reads associated with *Wolbachia* can affect the outcome of biodiversity comparisons. Although *Wolbachia* reads were removed in silico, the remaining sequences were insufficient in number to meaningfully characterize the bacterial community in this species, with insufficient reads defined as fewer than 15,284 reads (10% of the average reads per sample).

### 4.4. Symbionts Identified and Pest Management

The symbionts detected in this study, including *Wolbachia*, *Spiroplasma* and *Enterococcus*, can influence pests in multiple ways relevant to management. In our study, the presence of *E. mundtii* in *S. frugiperda* may play a role in modulating pesticide resistance, though this remains to be explored. Similar patterns have been reported in *P. xylostella*, where gut bacteria including *Enterococcus* have been shown to increase resistance to insecticides, particularly organophosphates [48, 124, 125], whereas *E. mundtii* can increase susceptibility to *Bacillus thuringiensis* (Bt) toxins [48]. Additionally, symbionts such as *Wolbachia* and *Spiroplasma* can influence the host reproduction, including cytoplasmic incompatibility and sex‐ratio distortion, potentially affecting pest population dynamics. The widespread occurrence of *Wolbachia* detected in this study may also have implications beyond reproductive manipulation. Previous studies have shown that *Wolbachia* infection can enhance resistance to insecticides such as fipronil and avermectin and may influence tolerance to other pesticides through effects on host detoxification pathways [135]. These *Wolbachia-*associated effects on host population dynamics and insecticide responses could potentially be exploited in future integrated pest management strategies, and monitoring their presence could help inform such programs. The endosymbionts identified here may also form the basis of future cross‐species transinfection attempts.

## 5. Conclusion

This study provides a survey of endosymbionts and other bacterial microbiota in key agricultural lepidopteran pests. Overall, endosymbionts were rare across the sampled species, but some species carried common infections, with *Wolbachia* being the most widespread endosymbiont. The broader microbiome composition appeared relatively consistent across populations and indicates the presence of *Enterococcus* and other bacteria that should be explored further. Our sampling focused primarily on grain‐associated Lepidoptera in Australia, with most specimens collected from Victoria. Different crops or regions may host lepidopteran populations with distinct endosymbiont or microbiome profiles, but at this stage we suspect that there is limited variation in the microbiota of these pest species. Expanding the sampling to include more host species could reveal additional infections or microbial taxa, particularly in species like *H. armigera* and *H. punctigera* where sampling was limited. The findings of this study provide a foundation for future research. Future experiments could include removing endosymbionts from sexually reproducing strains to assess potential fitness costs or transferring cytoplasmic incompatibility‐inducing or male‐killing endosymbionts between species to explore their potential for pest suppression. Investigating the gut microbiota of key pest species may also provide insights into microbial contributions to pesticide resistance. Such approaches could contribute to more sustainable pest management strategies, reducing the reliance on chemical pesticides.

## Author Contributions

Ary A. Hoffmann and Qiong Yang have contributed to the work equally and should be regarded as co‐first authors.

## Funding

This work was supported by Grains Research and Development Corporation (10.13039/501100000980; UOM1906‐002RTX and UOM2404‐006RT) and Hort Innovation (10.13039/501100000981; ST23002). Open access publishing facilitated by The University of Melbourne, as part of the Wiley ‐ The University of Melbourne agreement via the Council of Australasian University Librarians.

## Conflicts of Interest

The authors declare no conflicts of interest.

## Data Availability

All data and scripts used in this study are available in a University of Melbourne FigShare repository [136]: 10.26188/30171388.v1. Illumina short‐reads for 16S rRNA metabarcoding have been submitted to NCBI Sequence Read Archive under BioProject PRJNA1328949.
